# Exploring the hypolipidemic effects of bergenin from *Saxifraga melanocentra* Franch: mechanistic insights and potential for hyperlipidemia treatment

**DOI:** 10.1186/s12944-023-01973-2

**Published:** 2023-11-24

**Authors:** Li Zhang, Yingying Tong, Yan Fang, Jinjin Pei, Qilan Wang, Gang Li

**Affiliations:** 1 Qinghai Provincial Key Laboratory of Tibetan Medicine Research, Key Laboratory of Tibetan Medicine Research, Northwest Institute of Plateau Biology, Chinese Academy of Sciences, Xining, 810001 P. R. China; 2 Center for Mitochondria and Healthy Aging, College of Life Sciences, Yantai University, Yantai, 264005 P. R. China; 3 Qinba State Key Laboratory of biological resources and ecological environment, Province Key Laboratory of Bioresources, College of Bioscience and bioengineering, QinLing-Bashan Mountains Bioresources Comprehensive Development C. I. C, Shaanxi University of Technology, Hanzhong, 723001 Shaanxi China

**Keywords:** *Saxifraga melanocentra* Franch, Polyamide medium-pressure liquid chromatography, Bergenin, Hyperlipidemia

## Abstract

**Objective:**

The goal of this study was to explore the hypolipidemic effects of bergenin extracted from *Saxifraga melanocentra* Franch (*S. melanocentra*), which is a frequently utilized Tibetan medicinal plant known for its diverse bioactivities. Establishing a quality control system for black stem saxifrage is crucial to ensure the rational utilization of its medicinal resources.

**Methods:**

A one-step polyamide medium-pressure liquid chromatography technique was applied to isolate and prepare bergenin from a methanol extract of *S. melanocentra*. A zebrafish model of hyperlipidemia was used to investigate the potential hypolipidemic effects of bergenin.

**Results:**

The results revealed that bergenin exhibited substantial hypo efficacy in vivo. Specifically, bergenin significantly reduced the levels of triglycerides (TG), total cholesterol (TC), and low-density lipoprotein cholesterol (LDL-c) while simultaneously increasing high-density lipoprotein cholesterol (HDL-c) levels. At the molecular level, bergenin exerted its effects by inhibiting the expression of FASN, SREBF1, HMGCRα, RORα, LDLRα, IL-1β, and TNF while promoting the expression of IL-4 at the transcriptional level. Molecular docking analysis further demonstrated the strong binding affinity of bergenin to proteins such as FASN, SREBF1, HMGCRα, RORα, LDLRα, IL-4, IL-1β, and TNF.

**Conclusions:**

Findings indicate that bergenin modulates lipid metabolism by regulating lipid and cholesterol synthesis as well as inflammatory responses through signaling pathways associated with FASN, SREBF1, and RORα. These results position bergenin as a potential candidate for the treatment of hyperlipidemia.

## Introduction

Hyperlipidemia, characterized by a chronic disorder in lipid metabolism, represents a significant risk factor for the development of atherosclerosis, coronary heart disease, and other cardiovascular conditions [1]. The escalating incidence of hyperlipidemia in recent years, driven by dietary shifts toward high sugar and high fat consumption, poses a grave threat to human health. Consequently, the prevention and treatment of hyperlipidemia have emerged as pressing global concerns [2]. Zebrafish exhibit multiple adipose tissue depots, with neutral lipid droplets initially appearing in visceral adipocytes and accumulating as the zebrafish matures. These early zebrafish adipocytes resemble the white adipose tissue of mammals, consisting of numerous small lipid droplets, while mature zebrafish adipocytes typically possess a single large lipid droplet. Moreover, zebrafish store lipids in visceral, muscle, and subcutaneous adipocyte depots, displaying a conserved pattern of adipose tissue distribution and formation. Consequently, zebrafish serve as a suitable model for investigating hyperlipidemia and obesity, offering valuable insights into these conditions [3, 4].

Prolonged consumption of a high-fat diet can disrupt the equilibrium between lipid absorption and digestion, resulting in excessive fat and cholesterol deposition on the inner walls of blood vessels. This process leads to the formation of atheromatous plaques and hampers blood flow, ultimately giving rise to various severe cardiovascular diseases [5, 6]. Clinical recommendations for lipid control primarily involve dietary adjustments and exercise, along with the use of statins and fibrates [7, 8]. Although statins have demonstrated superior lipid-lowering effects, they can also lead to adverse effects such as elevated transaminase levels, increased blood glucose, and gastrointestinal symptoms [9, 10]. Hence, the reduction of adverse effects while effectively lowering lipid levels remains a significant concern in hyperlipidemia research.

*S. melanocentra* is a perennial herb belonging to the Sect. *Micranthae* of the *Saxifraga* genus within the Saxifragaceae family. It is widely recognized as a traditional Tibetan medicine called “Zhen Se Da E” and predominantly thrives in shrubs, meadows, and rocky crevices at altitudes ranging from 2800 to 4600 m. The plant’s distribution spans Qinghai, Gansu, Yunnan, and Tibet [11]. *S. melanocentra* was initially documented in the Ming Dynasty’s “Compendium of Materia Medica.“ As a common Chinese herb, it is renowned for its sweet and warming properties and is specifically employed for blood tonifying and eye ailment treatment. Tibetan medicine holds a prominent position within China’s rich medical history and remains a crucial component of traditional medicine. The concept of drug quality control in China can be traced back to the legendary tale of “Shennong tasted a hundred herbs.“ Ensuring the safety and efficacy of clinical drug application hinges upon the quality of medicinal substances. However, Tibetan medicine quality control presents certain challenges, including complex raw materials and significant batch-to-batch variations in ingredient composition [12]. These factors make it particularly difficult to guarantee the quality of Tibetan remedies. Consequently, the evaluation of indicator components with high content or high specificity has become a common practice in the current quality control system for Tibetan medicine [13]. Establishing a pertinent quality control system for *S. melanocentra* is therefore of paramount importance. Such a system would serve as a foundation for the development of new drugs and the rational utilization of medicinal resources derived from *S. melanocentra*.

Bergenin, an isocoumarin compound, has been incorporated into the Chinese Pharmacopoeia as a commercialized drug due to its notable antitussive properties [14, 15]. As a naturally derived product from plants, bergenin is widely accessible and cost-effective and holds promising prospects for development. In recent years, a multitude of studies have revealed its significant pharmacological effects, including anti-inflammatory, hepatoprotective, and antibacterial properties [16]. Clinically, bergenin is extensively used to treat chronic bronchitis, chronic gastritis, gastric ulcers, and duodenal ulcers [17]. It is important to highlight that the exploration of the hypo effects of bergenin is still at an early stage. Currently, research in this area remains limited, underscoring the significance of unraveling its hypo potential for the further development and utilization of its medicinal value.

Molecular docking technology plays a pivotal role in computer-aided drug design, facilitating the exploration of interactions between active ingredients and drug targets and aiding in the discovery and optimization of lead compounds [18, 19]. By employing a conformational search algorithm, the technology optimizes the conformation and positioning of the target protein‒ligand complex with small molecule compounds. Subsequently, the resulting conformations are scored to identify the compound with the most favorable binding effect [20]. The process of molecular docking can be divided into four key steps: protein and small molecule ligand preparation, binding site identification, conformational search of ligand compounds, and evaluation of docking results [21]. Presently, molecular docking finds widespread application in pharmaceutical research and development, as well as biomolecule design. Its utilization enhances the efficiency of virtual screening and contributes to reducing the costs associated with drug development.

In a previous study, our research group successfully isolated bergenin, the main compound from *S. melanocentra* using a two-step medium-pressure chromatographic approach involving polyamide and MCI GEL®CHP20P. This laid the groundwork for quality standard studies [22]. However, there has been no report on the application of one-step polyamide medium-pressure liquid chromatography (MPLC) for the isolation and purification of this key component. In this research, we purified bergenin, the primary active ingredient in *S. melanocentra*, from the plant using a one-step MPLC method with polyamide as the stationary phase. While previous investigations have explored on the cough-suppressant, antidiabetes, and hepatoprotective effects of bergenin, our study is the first to delve into its impact on lipid metabolism disorders. Notably, Rajesh Kumar et al. [23] indicated some hypolipidemic effects of bergenin, but the mechanism through which it reduces lipid elevation in the body has not been clearly elucidated. This study marks the first exploration of the effect of bergenin on lipid metabolism disorders. The present study seeks to offer a foundational and comprehensive understanding of bergenin’s hypolipidemic effect, shedding light on its potential therapeutic applications in the context of lipid disorders associated with *S. melanocentra*.

## Materials and methods

### Apparatus and chemicals

For the preparative MPLC workstation, we utilized two NP7000 prep-HPLC pumps, an NU3000 UV‒Vis detector, a 5 mL manual injector, and an LC workstation from Hanbon Science & Technology Co., Ltd., Huaian, Jiangsu, China. Before analysis, the samples were degassed using DGU-20A3R, and HPLC analysis was conducted using LC-16 A equipment equipped with a column thermostat and autosampler from Shimadzu Instruments Co., Suzhou, Jiangsu, China. ESI-MS data were acquired using a Waters QDa electrospray ionization (ESI) mass spectrometer from Waters Instruments Co., Milford, Massachusetts, USA. The ^1^ H and ^13^ C NMR spectra were recorded using a Bruker Avance 600 M*Hz* instrument (Bruker, Karlsruhe, Germany) with MeOH*d*_*4*_ as the solvent. The stereomicroscope used in the study was purchased from Nikon Corporation (Tokyo, Japan). Additionally, the multifunctional enzyme marker was obtained from Molecular Devices Corporation (Sunnyvale, USA).

Polyamide separation materials (100–200 mesh) were provided by Chengdu Quanlong Chemical Co. (Chengdu, China). ReproSil-Pur C18 AQ columns (4.6 × 250 mm, 5 μm) were obtained from Dr. Maisch & Co., Baden-Wurttemberg, Germany. The Click XIon column (4.6 × 250 mm, 5 μm) was supplied by ACCHROM Corporation (Beijing, China). Preparative methanol (CH3OH), acetonitrile (ACN), and high-performance liquid chromatography grade ACN were procured from China KELON Chemical Reagent Factory (Chengdu, China). Ultrapure water for HPLC was obtained using the Moore water purification station from deionized water (Chongqing, China). Egg yolk powder was purchased from Shanghai Yuanye Biotechnology Co., Ltd. (Shanghai, China), while *Artemia nauplii* was obtained from Shandong Binzhou Aijia Aquarium (Shandong, China). Nanjing Jiancheng Biological Company (Nanjing, China) provided triglyceride (TG), total cholesterol (TC), low-density lipoprotein cholesterol (LDL-c), and high-density lipoprotein cholesterol (HDL-c) test kits. The BCA protein assay kit and Oil Red O were supplied by Beyotime Institute of Biotechnology (Shandong, China) and Sigma‒Aldrich (St. Louis, MO, USA), respectively. Fenofibrate and methylcellulose were purchased from Aladdin (Shanghai, China). The SPARKeasy tissue cell RNA rapid extraction kit, SPARKscript II RT Plus Kit (with gDNA Eraser), and 2×SYBR Green qPCR Mix were all obtained from Shandong Sikejie Biotechnology Co., Ltd. (Shandong, China).

### Zebrafish and maintenance

Adult wild-type AB strain zebrafish were obtained from the China Zebrafish Resource Center (CZRC, Beijing, China) and individually housed in a culture system operating on a 14-h light and 10-h dark cycle. The culture system utilized deionized water, with controlled additions of NaHCO_3_ and NaCl to maintain a pH range of 7.0–8.0, conductivity between 450 and 550 μs, and a system temperature of 28.0 ± 0.5℃ using heating rods. Zebrafish embryos and larvae were incubated in fish water consisting of 5.0 mM NaCl, 0.17 mM KCl, 0.33 mM CaCl_2_, and 0.33 mM MgSO_4_ at a constant temperature of 28.5 °C [24]. All experimental procedures were conducted in compliance with the guidelines of the National Institutes of Health Guide for the Care and Use of Laboratory Animals, with approval number YDLL2022Z027.

### Sample preparation, purification, and purity analysis

The *S. melanocentra* herb was collected from Goluo Tibetan Autonomous Prefecture, Qinghai Province, in August 2016. The herb was identified as *S. melanocentra* by Prof. Lijuan Mei from the Northwest Institute of Plateau Biology, Chinese Academy of Sciences. An herb specimen was preserved with specimen number 0325734 in the Key Laboratory of Adaptation and Evolution of Plateau Biota, Chinese Academy of Sciences.

After harvesting, the *S. melanocentra* herb was dried in the shade. Approximately 200 g of the dried herb was weighed and soaked in a 4.0 L methanol solution at room temperature. The extraction process was performed three times, with one extraction per day. The resulting extracts were combined with approximately 12.0 L of methanol solution. The crude extract was then concentrated using a rotary evaporator under reduced pressure, yielding approximately 300 mL of extract. The crude extract was mixed with 100 g of polyamide and dried in a 40 °C oven. The polyamide-combined sample was crushed and sieved, and a total weight of 120.5 g was obtained.

Next, the dry mixture (60.0 g) was loaded into a small medium-pressure column (49 × 100 mm, Beijing Baosai Hope Biotechnology Co., LTD, Beijing, China) connected in series with a medium-pressure column (49 × 460 mm, Beijing Baosai Hope Biotechnology Co., LTD, Beijing, China) containing polyamide for sample loading. The elution was carried out using a water/acetonitrile system for 130 min. Within 100 min, a gradient elution from 0 to 100% acetonitrile was completed, followed by isocratic elution with 100% acetonitrile for 30 min. The elution process was performed at a constant flow rate of 57.0 mL/min, and the absorbance at 254 nm was monitored to track elution. This process was repeated twice at room temperature, resulting in a concentrated and dried target sample fraction weighing 1.36 g. Subsequently, 50.0 mg of Fr2 was dissolved in a mixture of methanol and water (70:30 *v/v*, 1.0 mL, 50 mg/mL) and passed through a 0.45 μm membrane.

To assess the purity of bergenin, ReproSil-Pur C18 AQ, XCharge C18, XAmide, and Click XIon analytical columns were utilized. Mobile phase A consisted of chromatographically pure water, while mobile phase B consisted of acetonitrile. Gradient elution using 5-35% acetonitrile, 5-30% acetonitrile, 95%-50% acetonitrile, and 95%-60% acetonitrile was performed on all four analytical columns for 60 min. The flow rate was set at 1.0 mL/min, and an injection volume of 5 μL was used. The detection wavelength for purity assessment was 254 nm.

### Establishment of a zebrafish hypo model

Twenty-day-old healthy zebrafish post fertilization (dpf) were randomly selected and transferred to six-well plates. They were incubated with fish water for the duration of the experiment. In the control group, zebrafish were fed 2 mg/fish/day *A. nauplii*. The high-fat model group, as well as the fenofibrate and bergenin groups, were fed ground *A. nauplii* (2 mg/fish/day) and egg yolk powder (1.5 mg/mL). Each group consisted of 10 zebrafish in a 10 mL culture system. The feeding regimen lasted for 7 consecutive days, with one feeding session lasting 6 h each day. After the 7-day feeding period, the control group continued to receive 2 mg/fish/day *A. nauplii*. The high-fat model group received 2 mg/fish/day *A. nauplii* and 1.5 mg/mL egg yolk powder. The fenofibrate and bergenin groups were also fed 2 mg/fish/day *A. nauplii* and 1.5 mg/mL egg yolk powder but with varying concentrations of fenofibrate and bergenin [25]. This feeding regimen was followed for 5 days, with 6 h of feeding per day. Fenofibrate and bergenin treatments lasted for 24 h.

### Acute toxicity test

Randomly selected healthy zebrafish embryos at 1 day post fertilization (dpf) were placed in six-well plates, with 30 embryos in each well. The embryos were divided into different groups: a blank control group treated with 0.1% DMSO and a bergenin group treated with varying concentrations of bergenin (0, 10, 33.3, and 100 μM). Each group had three parallel wells. The six-well plates were placed in a constant temperature light incubator set at 28 °C. The solution in the wells was changed every 24 h. Throughout the experiment, the mortality and hatching rate of the zebrafish embryos were carefully observed and recorded.

### Determination of blood lipid levels in zebrafish

One day prior to the conclusion of the experiment, the animals were subjected to an overnight fasting period. On the following day, five zebrafish were selected from each group. They were rinsed with cold PBS, and excess moisture was removed by blotting with filter paper. The wet weight of each zebrafish was recorded, and prechilled PBS at a volume nine times greater than the weight of the fish (weight (g): volume (mL) = 1:9) was added. The zebrafish specimens were mechanically homogenized on ice and then centrifuged at 12,000 g/min for 15 min [26]. The resulting supernatant was collected, and the levels of triglycerides (TG, Production No. 20,220,928), total cholesterol (TC, Production No. 20,220,817), low-density lipoprotein cholesterol (LDL-c, Production No. 20,220,519), and high-density lipoprotein cholesterol (HDL-c, Production No. 20,220,707) were determined using commercially available assay kits (produced by Nanjing Jiancheng Biological Engineering Research Institute Co. (Jiangsu, China)), following the instructions provided with the kits.

### Observation of overall lipid accumulation in zebrafish

After reaching the experimental endpoint, the zebrafish were subjected to a 24-h fasting period. From each group, eight zebrafish were randomly selected for oil red O staining. The larvae were anesthetized using a 0.016% tricane solution, washed twice with PBS, and then fixed overnight at 4 °C in freshly prepared 4% paraformaldehyde. The following day, the fixed zebrafish were removed from the fixative, washed twice with PBS, and permeabilized by incubating them in a 60% isopropanol solution for 30 min. The 60% isopropanol solution was then removed, and a freshly prepared 0.5% oil red O solution was added to the zebrafish, which were incubated in the dark for 3 h. After incubation, the oil red O staining solution was discarded, and the zebrafish were gently shaken for 3 min in 60% isopropanol to wash away the background staining [27]. PBS was then added, and the zebrafish were shaken gently three times for 10 min each time until the signal became clearly visible [27]. The accumulation of lipids in the zebrafish was observed and documented under a stereomicroscope.

### Zebrafish behavioral assays

The zebrafish that received drug treatment were placed individually in separate wells of a 24-well plate containing 1 mL of fish water. A video camera (C922; Logitech, Shanghai, China) positioned above the plate recorded the movements of the zebrafish. SMART 3.0 software (Panlab Harvard Apparatus, MA, USA) was used to analyze the recorded videos and quantify the movement distance (in mm) of each fish [28]. For this experiment, both the 15-min movement distance and the 60-second movement trajectory of the zebrafish were recorded and analyzed.

### Real-time quantitative PCR

At the end of the experiment, animal samples were collected, and total RNA (Production No. AC0202-A, Shandong Sikejie Biotechnology Co., Ltd, China) was extracted from zebrafish embryos using a specific kit. The extracted RNA was then reverse transcribed to obtain cDNA templates. The expression levels of several target genes (FASN, LPL, SREBF1, HMGCRα, LDLRα, RORα, IL-1β, IL-4, and TNF) were measured using real-time quantitative PCR (qRT‒PCR) [29]. The sequences of primers used for qRT‒PCR amplification of the target genes are provided in Table 1.

**Table 1:** Each primer gene sequence

Gene	Forward primer	Reverse primer
β-Actin	ATGGATGAGGAAATCGCTGCC	CTCCCTGATGTCTGGGTCGTC
FASN	ACGGCAATGTCACCCTACTG	ATGCGAAGGTTTAGCCCTCT
LPL	CGCTCATGTTGCAGGAATCG	ACCGGCCTTTGAATCCCAAT
SREBF1	ACTCTGAAACCGGACGTGAC	TACGGTTGATGGGCAGCTTT
HMGCRα	CCCTCATTGAACCGCACTGTA	GGTAGCCACAATGACTTCCCA
LDLRα	TGGCTATTTTTACCCTCAGAGACA	TGCTGAAGAACTGACCTCCG
RORα	TATGGACCGCCGAGCTTAATG	GCGGTCAATCAGGCAGTTCT
IL-1β	GTGGACTTCGCAGCACAAAA	CACGTTCACTTCACGCTCTTG
IL-4	TGCAGCATATACCGGGACTGG	TCTTATGTCCTTTGAGCCGAGT
TNF	CAAATCACCACACCTTCAGCTTC	CACACCGCCAACCCATTTCA

### Molecular docking analysis

Molecular docking methods were employed to evaluate the interactions between biomolecular complexes, such as receptors and drugs. Protein crystal structures (PDB ID: 2PX6, 1AM9, 6E7K, 1HW8, 1N83, 4NE9, 4YDY, 1ITB, and 2E7A) representing FASN, SREBF1, LPL, HMGCRα, RORα, LDLRα, IL-4, IL-1β, and TNF were obtained from the Protein Data Bank (https://www.rcsb.org/) database. The receptors were prepared by removing water molecules and ions using PyMOL software. Polar hydrogen atoms and Kollman charges were then added, and the active sites of the proteins were identified and defined. The default parameters were used for the minimization process employing the Lamarckian genetic algorithm [30]. Following the docking procedure, 100 docking conformations were generated, and the best-scoring conformation was selected for further analysis.

### Statistical analyses

The statistical analysis was conducted using SPSS 22.0 software (SPSS, Chicago, IL, USA) to determine the significance of mean differences between groups using one-way ANOVA. GraphPad Prism 8.0.1 software (GraphPad, San Diego, CA, USA) was utilized for data visualization. All experimental results are presented as the mean ± standard deviation (SD) from three to six independent experiments. Statistical significance was indicated as *P < 0.05, representing a statistically significant difference, and **P < 0.01, indicating a highly statistically significant difference.

## Results and discussion

### Bergenin preparative isolation and purity analysis

**Fig. 1:**
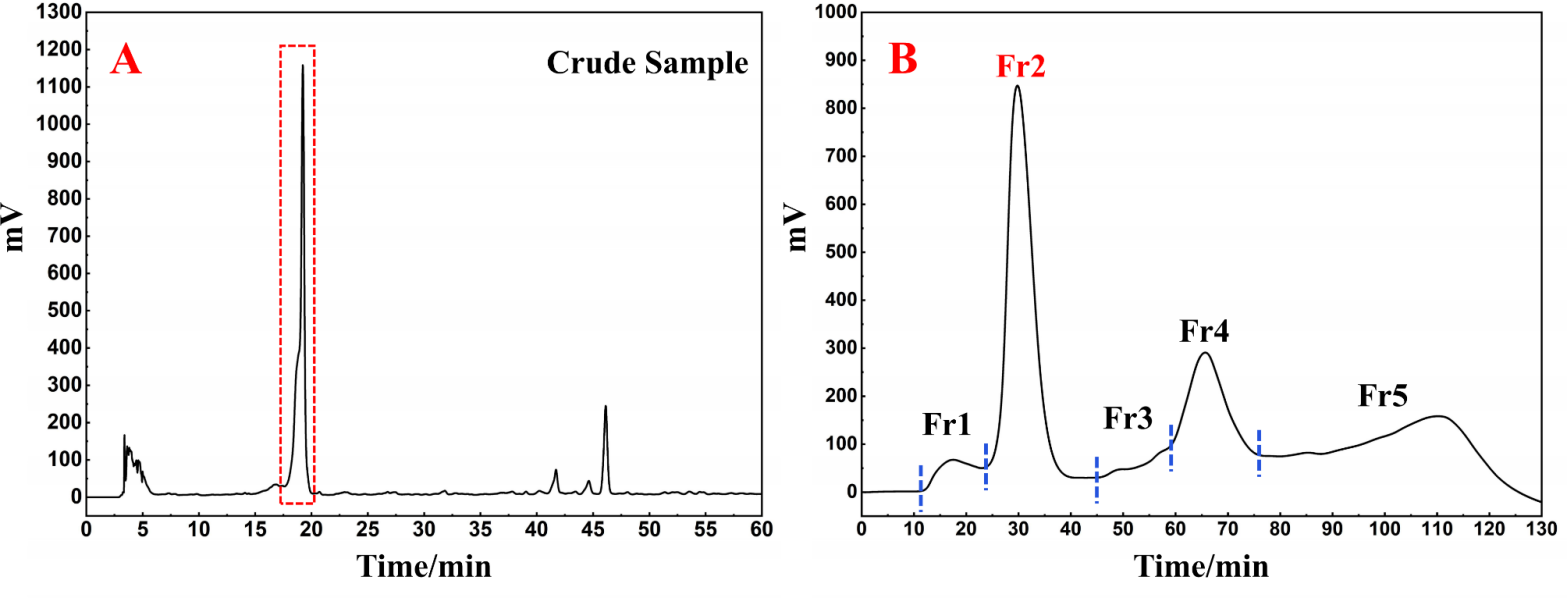
Analytical chromatogram on a Reprosil-Pur C18 AQ analytical column of the *S. melanocentra* methanol extract (**A**) and separation chromatogram of the *S. melanocentra* polyamide mixture (**B**)

Considering the limited research on the chemical composition of *S. melanocentra*, the concentrated methanolic extract was initially analyzed using an analytical column, ReproSil-Pur C18 AQ. The chromatogram depicting the analysis is presented in Fig. 1A. As observed in Fig. 1A, an index component was detected in the extract. However, the ReproSil-Pur C18 AQ analytical column did not provide optimal separation for the index component, resulting in a peak with poor symmetry and a nonideal parcel-like shape (highlighted by the red box in Fig. 1A). This indicates that the ReproSil-Pur C18 AQ column may not be the most suitable choice for achieving high-purity separation of the index component in *S. melanocentra*. Regarding the effectiveness of polyamide chromatography in separating flavonoids, phenolics, quinones, and polar compounds, such as alkaloids and steroids, from nonpolar compounds [31, 32], it was deemed a more suitable option. Furthermore, the large separation volume of the polyamide column makes it appropriate for preparative separation. Subsequently, the mixed sample was crushed, sieved, and weighed, resulting in a total sample weight of 120.5 g. Based on calculations, the yield of the total sample was determined to be 10.3%, equivalent to 20.5 g.

Polyamides are macromolecular substances that result from the polymerization of amides and contain multiple amide groups in their molecular structure [33]. In this study, a cost-effective MPLC technique utilizing polyamide was employed to isolate bergenin from the methanol extract of *S. melanocentra*. During the experimental process, a preparative liquid chromatograph equipped with a high-performance medium-pressure column (49 × 460 mm) filled with polyamide was employed. Additionally, a small medium-pressure column (49 × 100 mm) was used to facilitate the loading of a mixture comprising dried polyamide and the sample (60.0 g) for the preparation of dry-loaded samples. The resulting chromatogram of the preparation is depicted in Fig. 1B. Following two enrichment cycles using an acetonitrile-water eluent, five fractions (Fr1, Fr2, Fr3, Fr4, and Fr5) were collected. The results demonstrated the efficacy of polyamide MPLC in achieving the efficient preparation of active ingredients, thus highlighting it as a favorable method for sample separation.

**Fig. 2:**
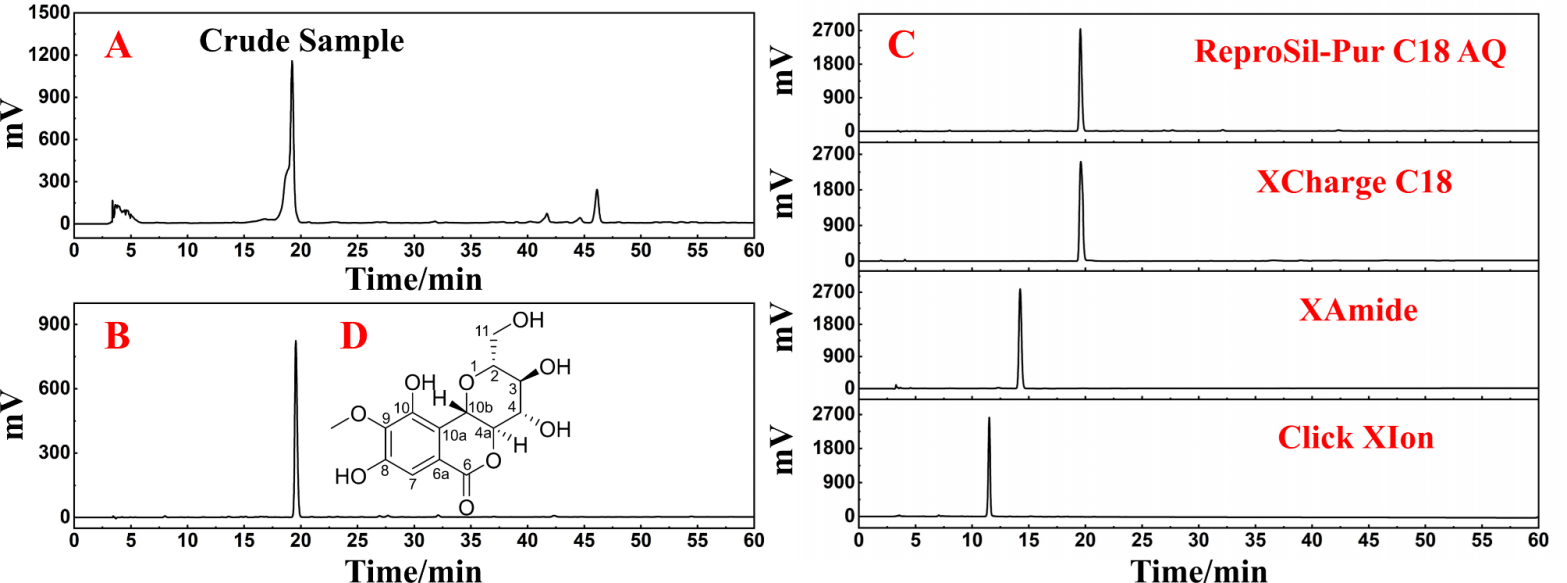
HPLC analysis of the *S. melanocentra* methanol extract (**A**), Fr2 (**B**) on the Reprosil-Pur C18 AQ analytical column, purity analysis comparison figures of the isolated main fraction Fr2 on the ReproSil-Pur C18 AQ, XCharge C18, XAmide, and Click XIon analytical column (**C**) and chemical structures of the isolated compound (**D**)

After two repeated separations, the collected target fraction was combined and subjected to concentration, resulting in the obtainment of 1.36 g of the desired sample (Fr2) with a recovery rate of 6.8%. The HPLC chromatograms of the methanol extract of *S. melanocentra* and the Fr2 sample were then analyzed and compared using the ReproSil-Pur C18 AQ analytical column. The corresponding results are presented in Fig. 2A and B. The analysis showed that the primary compound present in the crude extract was significantly enriched in the Fr2 fraction. Notably, Fr2 exhibited a single peak with excellent symmetry when subjected to analysis on the ReproSil-Pur C18 AQ analytical column, indicating a sufficiently high level of purity. These findings suggest that polyamide and C18 columns possess complementary selectivity and can be effectively combined for the separation of bergenin.

To further validate the purity of Fr2, additional analysis was conducted using an RP column (XCharge C18) and HILIC columns (XAmide and Click XION). The corresponding analytical chromatogram is presented in Fig. 2C. Upon examination of these chromatographic columns, it was observed that the main component Fr2 exhibited a purity level exceeding 99%. Retention mechanisms in reversed-phase liquid chromatography (RPLC) and hydrophilic interaction liquid chromatography (HILIC) differ due to varying principles [34, 35]. The ReproSil-Pur C18 AQ analytical column is bonded with ultrapure silica gel and specifically designed for hydrophilic and polar compounds, offering improved retention and selectivity. It demonstrates excellent pH stability and batch repeatability. The XCharge C18 analytical column employs electrostatic control technology, allowing for the regulation of hydrophobicity and electrostatic properties on the material surface by balancing positive and negative charges. This enables effective separation of analytes. The XAmide column, featuring a neutral amide-bonded phase, exhibits remarkable hydrophilicity, thereby overcoming issues related to the acidity and inhomogeneity of silanol groups found on pure silica gel surfaces. It provides an alternative to commonly used amino and silica gel columns by avoiding potential electrostatic effects. The XAmide column facilitates good peak shapes and separations for various strongly polar compounds [36]. Moreover, the Click XIon analytical column possesses surface hydrophilicity and controllable surface electrostatic effects, allowing for the separation of highly polar and charged hydrophilic compounds [37, 38]. Considering the complementary selectivity of the RP columns (ReproSil-Pur C18 AQ and XCharge C18) and HILIC columns (XAmide and Click XIon), the purity of the main component Fr2 from *S. melanocentra* was thoroughly confirmed.

The obtained ESI-MS, ^1^ H NMR, and ^13^ C NMR spectra were compared with published literature to determine the structure of the main compound, Fr2 (Fig. 2D). The structural identification of Fr2 is presented in Fig. 3A-D of the supplementary information. Based on the spectral data, the target compound was identified as bergenin. Compound Fr2 (bergenin) was obtained as a white powder weighing 1.36 g. The ESI-MS spectrum exhibited peaks at *m/z* 351.19 ([M + Na]^+^) and *m/z* 327.16 ([M − H]^−^), while the calculated mass for C_14_H_16_O_9_ was *m/z* 328.08. The ^1^ H NMR spectrum (600 M*Hz*, MeOH-*d*_*4*_) displayed signals at 7.08 (1 H, s, H-7), 4.95 (1 H, d, *J* = 10.5 *Hz*, H-10b), 4.04 (2 H, m, H-4a, H-4), 3.90 (3 H, s, H-12), 3.80 (1 H, t, *J* = 7.6 *Hz*, H-2), 3.68 (2 H, m, H-11), and 3.42 (1 H, m, H-3). The ^13^ C NMR spectrum (151 M*Hz*, MeOH-*d*_*4*_) exhibited peaks at 165.8 (C-6), 152.4 (C-8), 149.5 (C-10), 142.3 (C-9), 119.5 (C-6a), 117.3 (C-10a), 111.0 (C-7), 83.1 (C-2), 81.5 (C-4a), 75.6 (C-4), 74.3 (C-10b), 71.9 (C-3), 62.7 (C-11), and 60.9 (C-12). The obtained ESI-MS, ^1^ H NMR, and ^13^ C NMR data were consistent with the literature data for bergenin [39].

**Fig. 3:**
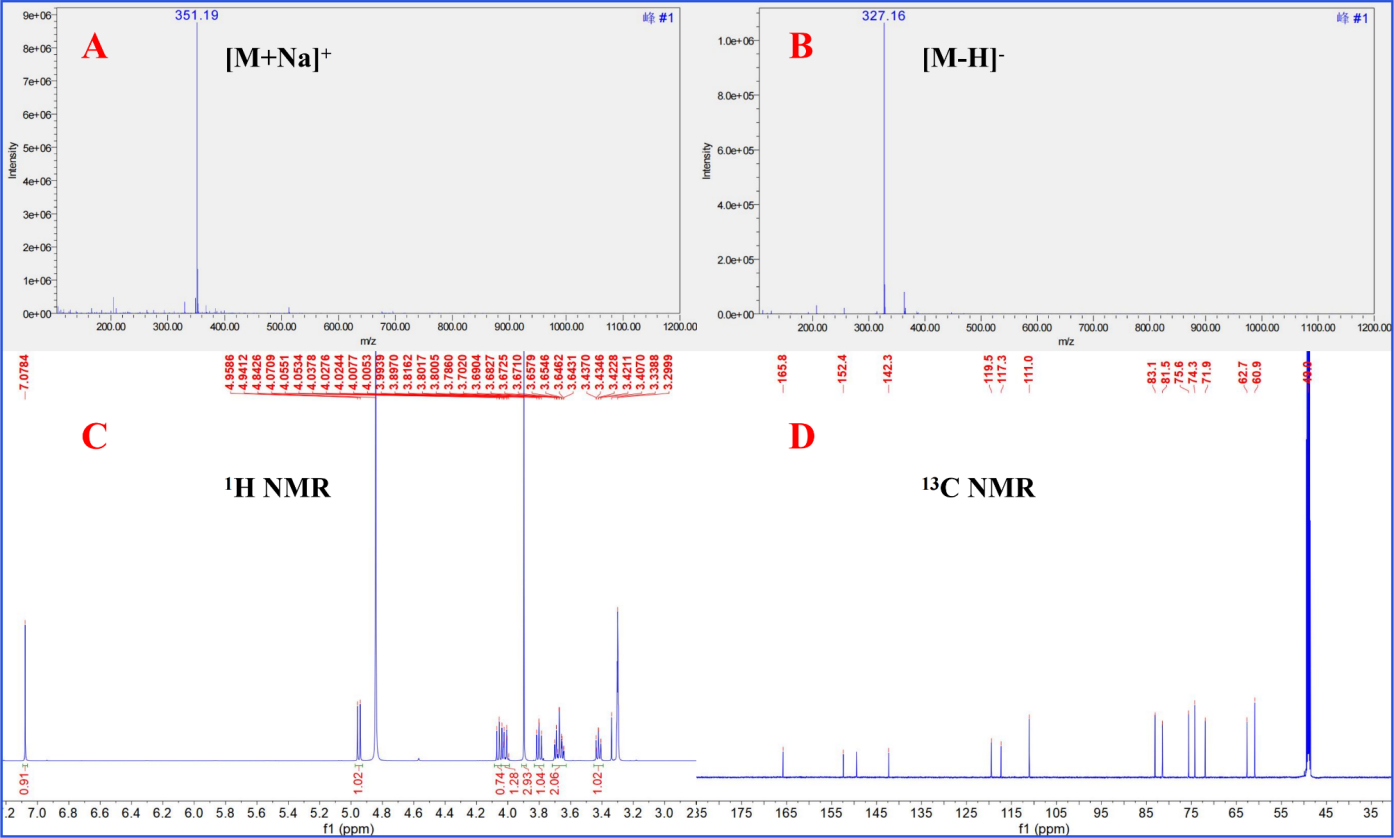
ESI-MS and NMR of the isolated compound Fr2 (bergenin). $+$ESI mass of compound Fr2 (**A**), $-$ESI mass of compound Fr2 (**B**), ^1^ H NMR spectrum (600 M*Hz*) of compound Fr2 (**C**), and ^13^ C NMR spectrum (151 M*Hz*) of compound Fr2 (**D**)

### Pharmacological effects of bergenin against hyperlipidemia based on the zebrafish model

#### Acute toxicity test results of bergenin

**Fig. 4:**
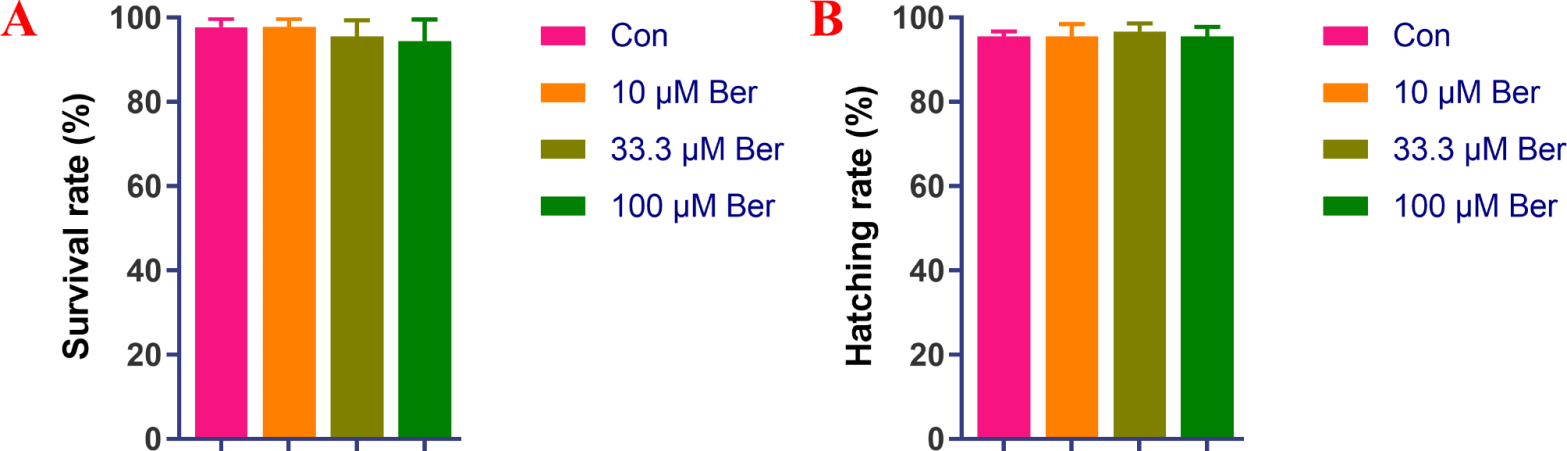
Effect of different concentrations of bergenin on embryonic mortality and hatching rate of zebrafish. Figure **A** shows the survival rate, and Figure **B** shows the hatching rate

The outcomes of the acute toxicity study revealed that petroselin at various concentrations (0, 10, 33.3, and 100 μM) did not induce toxicity in zebrafish. The mortality rate of zebrafish larvae was assessed at different bergenin concentrations (0, 10, 33.3, and 100 μM), as depicted in Fig. 4A and B. Zebrafish were deemed deceased if no visible activity was observed in the petri dish during the experiment and touching the tail did not elicit a response. There was no notable variation in the mortality rate relative to the exposure concentration of zebrafish after 96 h of exposure to petroselin in the experiment. Moreover, the survival and hatching rates of zebrafish larvae in the 0, 10, 33.3, and 100 μM bergenin treatment groups consistently exceeded 80%. Based on these results, doses of 5 μM and 15 μM were chosen for subsequent experiments, ensuring that they remained below the concentration of 33.3 μM.

#### Effect of bergenin on lipid levels in zebrafish

**Fig. 5:**
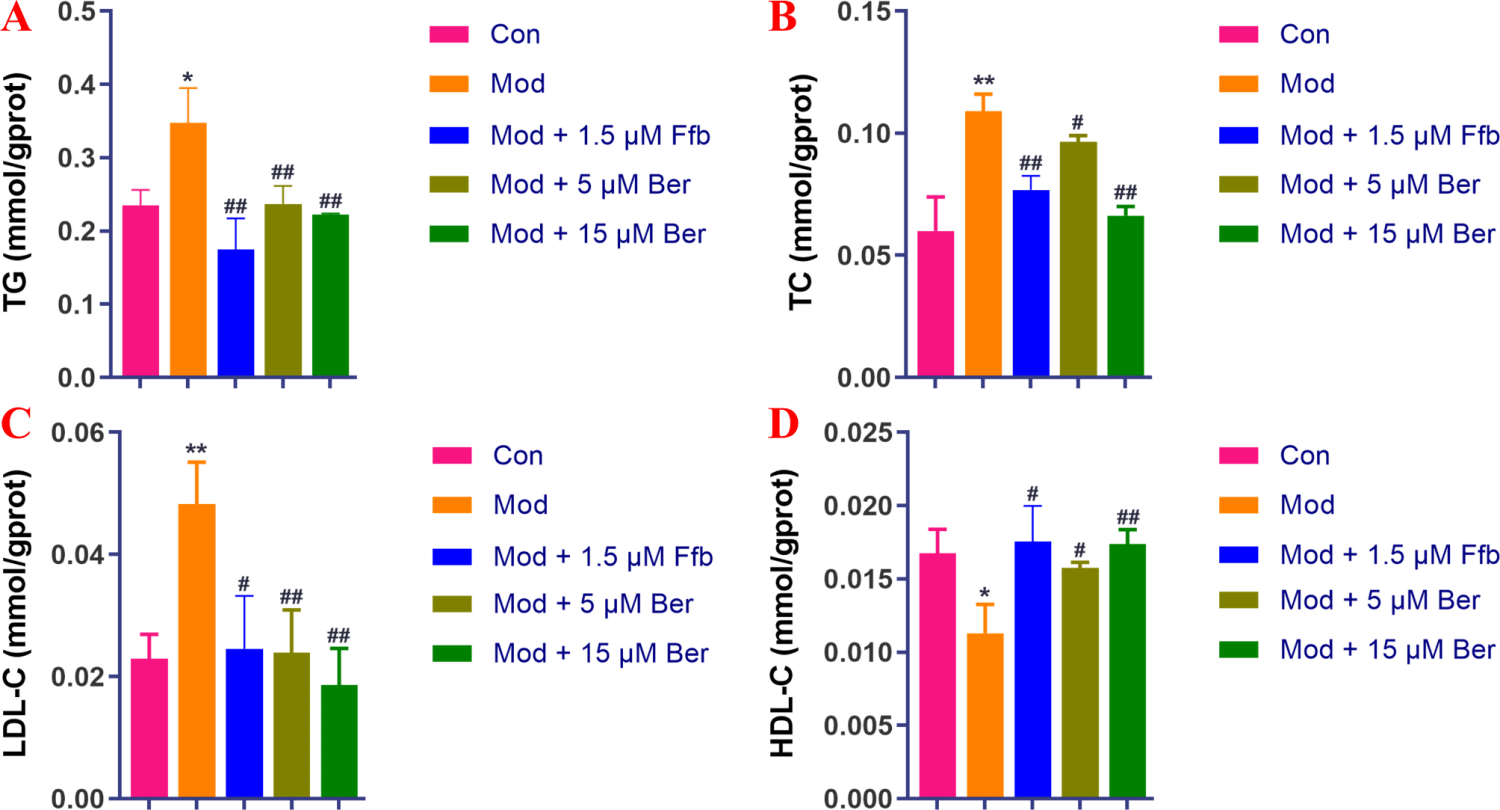
Effect of bergenin on blood lipid levels in high-fat zebrafish. Panels **A**, **B**, **C**, and **D** represent the contents of TG, TC, LDL-c, and HDL-c, respectively. Ffb: fenofibrate; Ber: bergenin. Values are presented as the mean ± SD. ^*^P < 0.05, ^**^P < 0.01 vs. the control group; ^#^*P* < 0.05, ^##^*P* < 0.01 vs. the model group

In our investigation, we explored the hypolipidemic impact of bergenin on a zebrafish model of hyperlipidemia induced by a high-fat diet. Figure 5 A-D depict the lipid profile of zebrafish larvae. In the model group, there were significantly heightened levels of TG (*P* < 0.05), TC (*P* < 0.01), and LDL-c (*P* < 0.01), accompanied by significantly reduced levels of HDL-c compared to the blank control group (*P* < 0.05). However, in the bergenin-treated group, there were significant reductions in the levels of TG, TC, and LDL-c, coupled with a significant increase in HDL-c. The changes in the levels of TG, TC, LDL-c, and HDL-c exhibited a dose-dependent relationship when compared with the model control group. Specifically, as the concentration of bergenin increased to 15 μM, the levels of TG, TC, and LDL-c in zebrafish decreased by 12.49% (*P* < 0.01), 4.27% (*P* < 0.01), and 2.96% (*P* < 0.01), respectively. Furthermore, the HDL-c levels in zebrafish increased by 0.29% (*P* < 0.01) when the concentration of bergenin was elevated to 15 μM. Notably, the higher concentration of bergenin exhibited a superior therapeutic effect compared to fenofibrate in reducing TC and LDL-c levels. Hyperlipidemia is a common disorder characterized by elevated plasma TG, TC, and LDL-c levels as well as decreased HDL-c levels. LDL-c plays a vital role in transporting TC to peripheral tissues, but excessive LDL-c and TC can lead to their deposition on arterial walls, resulting in endothelial cell damage, increased vascular permeability, and the development of atherosclerosis. This process involves the entry of plasma lipoproteins into the arterial intima, the recruitment and differentiation of monocytes into macrophages, and the formation of foam cells, which ultimately contribute to the formation of fibrous and atheromatous plaques, leading to cardiovascular diseases such as atherosclerosis [40, 41]. On the other hand, HDL-c facilitates the transport of excess cholesterol from peripheral tissues to the liver for metabolism and exhibits potent anti-inflammatory effects, thereby playing a protective role against atherosclerosis [42]. Lowering TG, TC, and LDL-c levels while increasing HDL-c levels is crucial for the prevention and treatment of cardiovascular diseases. The findings indicate that bergenin possesses effective regulatory properties on lipid metabolism disorders by reducing TG, TC, and LDL-c levels and increasing HDL-c levels. These results highlight the potential of bergenin in managing lipid disorders and preventing cardiovascular diseases.

#### Effect of bergenin on overall lipid accumulation in zebrafish

**Fig. 6:**
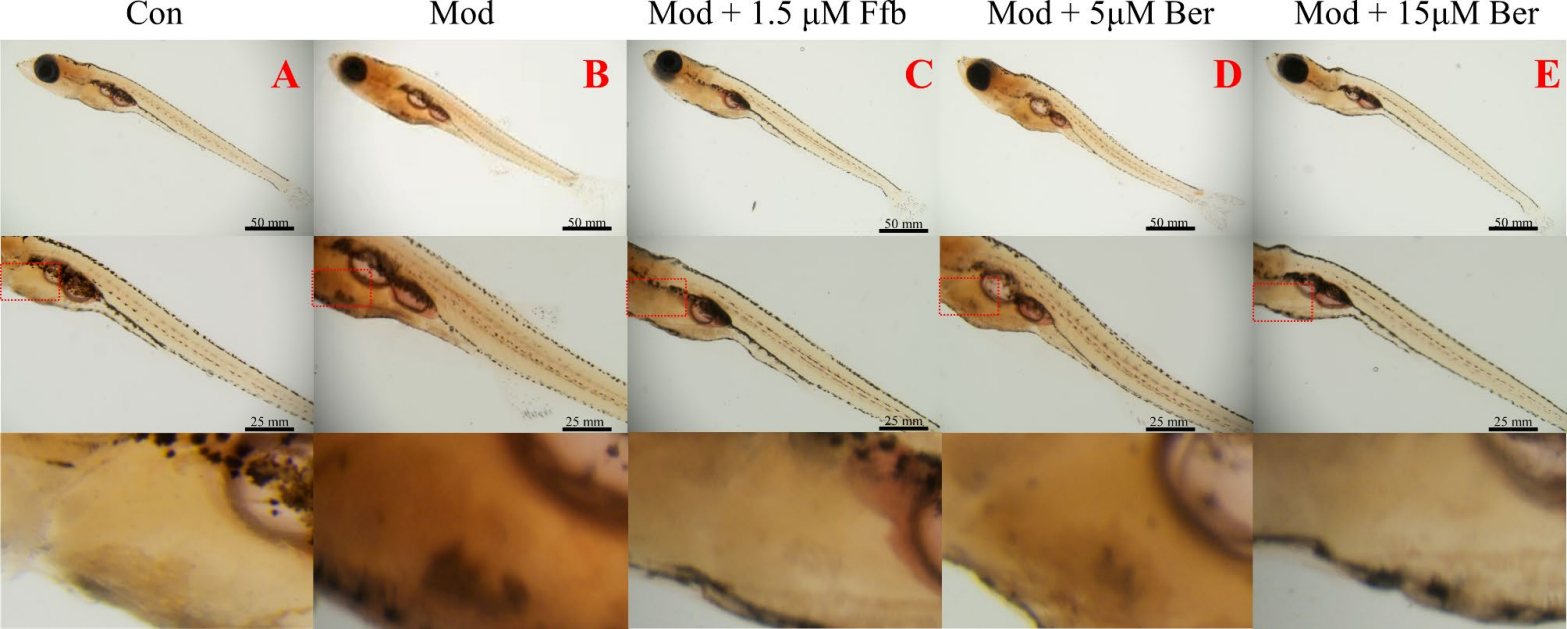
The effect of bergenin on overall lipid accumulation in high-fat zebrafish. (**A**): HE staining results in the control group; (**B**): HE staining results in the model group; (**C**) HE staining results in the 1.5 μM fenofibrate group; (**D**) and (**E**): HE staining results in the 5 and 15 μM bergenin groups. The upper figure represents a magnification of 20×, the middle figure represents a magnification of 40×, and the lower figure is a partial magnification of the zebrafish liver area (red box). Ffb: fenofibrate; Ber: bergenin. Values are presented as the mean ± SD. ^*^P < 0.05, ^**^P < 0.01 vs. the control group; ^#^*P* < 0.05, ^##^*P* < 0.01 vs. the model group

The solubility of Oil Red O dye is higher in tissues and cells compared to its original solvent, enabling its binding to TG and the formation of lipid droplets with an orange‒red appearance. Juvenile zebrafish, characterized by relatively transparent bodies with minimal melanin accumulation, allow for clear observation of lipid accumulation status under a microscope after staining. The staining results are depicted in Fig. 6A-E. In the control group, minimal Oil Red O staining was observed in the liver and tail vessels of zebrafish juveniles. In contrast, the high lipid model group displayed prominent orange‒red staining in these areas, indicating successful establishment of the zebrafish hyperlipidemic model with the accumulation of large lipid droplets. Lipid droplets were significantly reduced in the positive control (fenofibrate) and bergenin-treated groups compared to the model group. Remarkably, orange‒red lipid droplets in the liver and caudal vessels of zebrafish larvae decreased significantly and appeared lighter in color with increasing doses of petroselin. Notably, the high concentration of bergenin (15 μM) demonstrated a more pronounced lipid-lowering effect than fenofibrate. The effect of 15 μM petroselin in reducing lipid deposition in zebrafish was comparable to that of lipid staining in zebrafish in the blank control group. These findings provide additional evidence that bergenin effectively mitigates lipid deposition in the liver and caudal vessels of high-fat zebrafish larvae, highlighting its potential as a lipid-lowering agent.

#### Effects of bergenin on the behavior of high-fat zebrafish

**Fig. 7:**
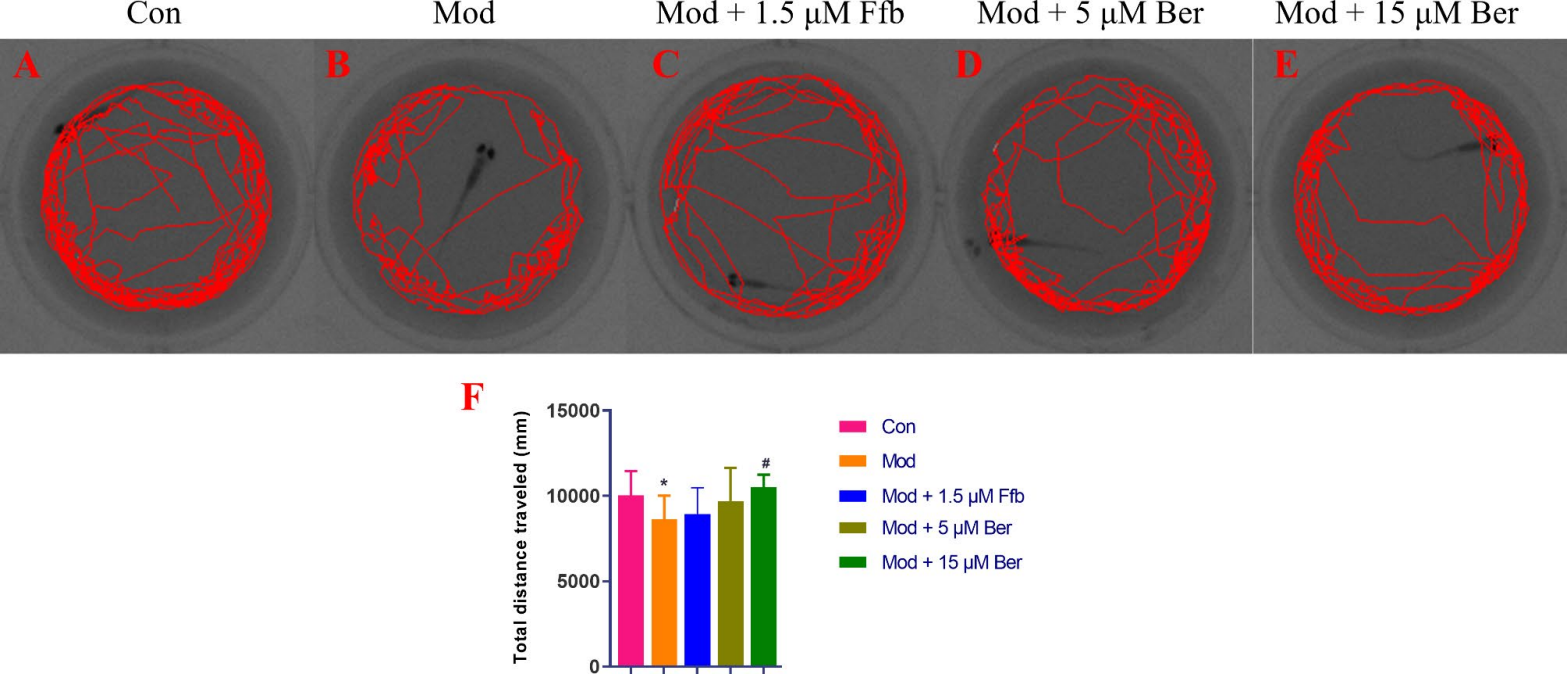
Effect of bergenin on the behavior of high-fat zebrafish. Figures **A**, **B**, **C**, **D**, and **E** represent the behavioral trajectory of each group of zebrafish, and **F** represents the total distance traveled by zebrafish in 15 s. Ffb: fenofibrate; Ber: bergenin. Values are presented as the mean ± SD. ^*^P < 0.05, ^**^P < 0.01 vs. the control group; ^#^*P* < 0.05, ^##^*P* < 0.01 vs. the model group

Excessive lipid accumulation in the body adversely affects physical activity. The consumption of a high-fat diet disrupts the secretion of muscle factors and interferes with glucolipid metabolism, leading to obesity and impairing overall body movement. The utility of behavioral responses as indicators of motor neuron function in organisms has been established [43]. Moreover, studies have investigated the impact of lipid-lowering compounds on motor behavior in the zebrafish hyperlipidemic model system [44, 45]. To evaluate the effects of different treatments, behavioral tests were conducted on zebrafish in various experimental groups, and the findings are illustrated in Fig. 7A-F. Zebrafish in the high-fat model group exhibited a significantly reduced total swimming distance compared to the blank control group, indicating compromised activity levels. Compared to the model group, zebrafish treated with bergenin (5 μM) and fenofibrate (1.5 μM) showed increased swimming distances, although the differences were not statistically significant. However, zebrafish in the 15 μM bergenin group demonstrated a significant increase in total swimming distance (*P* < 0.05). These results suggest that bergenin has the potential to enhance the impaired behavior of zebrafish caused by a high-fat diet. Furthermore, the concentration of bergenin showed a positive correlation with the observed improvement in behavioral performance.

### Mechanism of action of bergenin against hyperlipidemia

Cholesterol, a vital lipid in living organisms, is predominantly taken up by macrophages, serving as a major pathway for its uptake in the body. Macrophage lipid metabolism disorders encompass irregular cholesterol uptake and conversion, aberrant cholesterol reversal and extracellular transport, and abnormal expression of genes involved in lipid metabolism regulation [46, 47]. To explore the underlying mechanism of the effect of bergenin on improving hyperlipidemia, the present study employed qRT‒PCR to assess the expression levels of various genes, including FASN, SREBF1, LPL, HMGCRα, RORα, LDLRα, IL-4, IL-1β, and TNF, across different experimental groups. FASN, a complex enzyme system consisting of seven enzymes encoded by the same gene, predominantly resides in liver tissue. Its activity directly influences the liver’s capacity for synthesizing fatty acids, playing a critical role in regulating fat accumulation in animals [42]. Acting as an upstream transcription factor, SREBF1 governs the expression of FASN and exerts an impact on lipid aggregation by regulating fatty acid synthesis, thereby promoting lipogenesis and maintaining lipid homeostasis [42]. Lipoprotein lipase (LPL) serves as a pivotal rate-limiting enzyme in lipid catabolism, facilitating the breakdown of triglycerides into free fatty acids and mono-fatty acid triglycerides to provide the body with energy through oxidative breakdown [48]. 3-Hydroxy-3-methylglutaryl coenzyme A reductase (HMGCR), a vital component of human lipid synthesis, plays a crucial role in regulating cholesterol synthesis and metabolism. As cholesterol synthesized in the liver serves as the primary source of cholesterol in the body, HMGCR acts as the rate-limiting enzyme catalyzing the conversion of 3-hydroxy-3-methylglutaryl coenzyme A to mevalonate during cholesterol synthesis. Inhibiting HMGCR activity in the liver can reduce cholesterol synthesis and help regulate lipid metabolism disorders [49]. Low-density lipoprotein receptor alpha (LDLRα) is a hepatic receptor responsible for mediating the endocytosis of cholesterol-rich LDL particles, thereby maintaining optimal LDL plasma levels. Retinoic acid receptor-related orphan receptor alpha (RORα) plays a pivotal role in maintaining lipid metabolism homeostasis by regulating the transcriptional activity of target genes involved in triacylglycerol metabolism. Additionally, RORα serves as a negative regulator of inflammation [50]. Interleukin-4 (IL-4) is a growth factor secreted by T cells that functions as an anti-inflammatory factor with immunosuppressive effects on the body [51]. Interleukin-1 beta (IL-1β) and tumor necrosis factor alpha (TNF-α) are essential cytokines in the body and are primarily produced by active monocytes and vascular endothelial cells. They exert diverse biological effects and play a role in various pathological changes associated with inflammatory lesions in the body [52, 53].

**Fig. 8:**
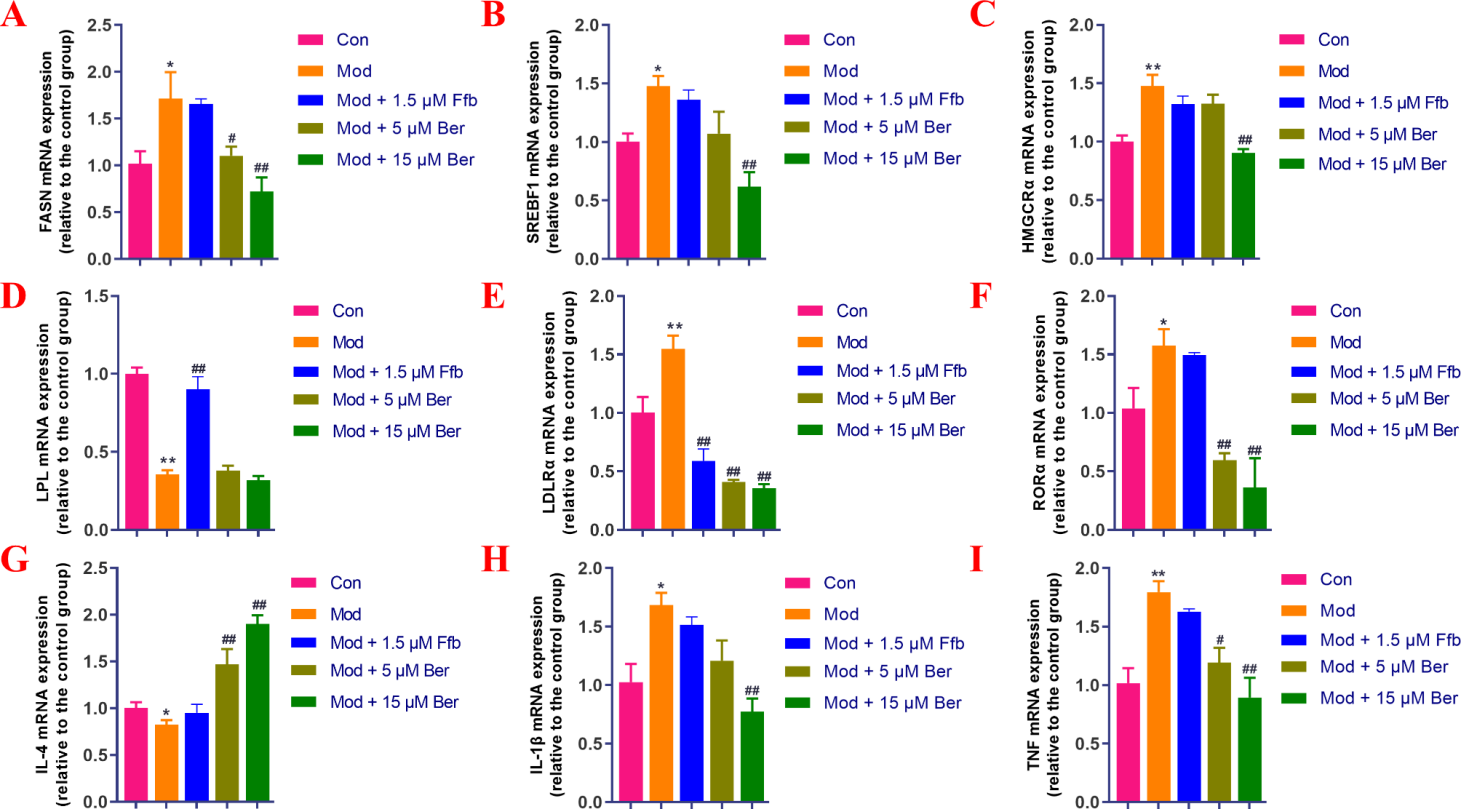
Bergenin impacts the gene expression of FASN, SREBF1, HMGCRα, LPL, LDLRα, RORα, IL-4, IL-1β, and TNF mRNA in high-fat diet-fed zebrafish. FASN mRNA expression (**A**), SREBF1 mRNA expression (**B**), HMGCRα mRNA expression (**C**), LPL mRNA expression (**D**), LDLRα mRNA expression (**E**), RORα mRNA expression (**F**), IL-4 mRNA expression (**G**), IL-1β mRNA expression (**H**), and TNF mRNA expression (**I**). Ffb: fenofibrate; Ber: bergenin. Values are presented as the mean ± SD. ^*^P < 0.05, ^**^P < 0.01 vs. the control group; ^#^*P* < 0.05, ^##^*P* < 0.01 vs. the model group

The results of the experiment are presented in Fig. 8A-I. In comparison to the blank control group, the high-fat model group exhibited significantly increased expression levels of FASN, HMGCRα, SREBF1, LDLR, RORα, IL-1β, and TNF mRNA. Conversely, the expression levels of LPL and IL-4 mRNA were significantly downregulated. In the bergenin treatment group, compared to the model group, the expression levels of SREBF1, HMGCRα, and IL-1β mRNA were reduced, with statistically significant differences observed under 15 μM bergenin administration. The expression levels of FASN, RORα, LDLRα, and TNF mRNA were significantly downregulated under 5 μM and 15 μM bergenin administration, showing statistically significant differences that were concentration dependent. After bergenin treatment, the expression level of IL-4 mRNA was significantly upregulated. However, bergenin did not affect the expression level of LPL mRNA. These experimental findings indicate that bergenin can modulate lipid synthesis, cholesterol metabolism, and the abnormal expression of inflammation-related genes. The results suggest that bergenin primarily reduces lipid deposition by inhibiting lipid synthesis rather than promoting lipolysis via the SREBF1 and FASN-related signaling pathways. Additionally, bergenin ameliorates abnormal lipid metabolism by inhibiting RORα expression, thereby regulating HMGCRα, LDLRα, IL-4, IL-1β, and TNF expression to inhibit cholesterol synthesis and mitigate the body’s inflammatory response.

### Molecular docking

**Fig. 9:**
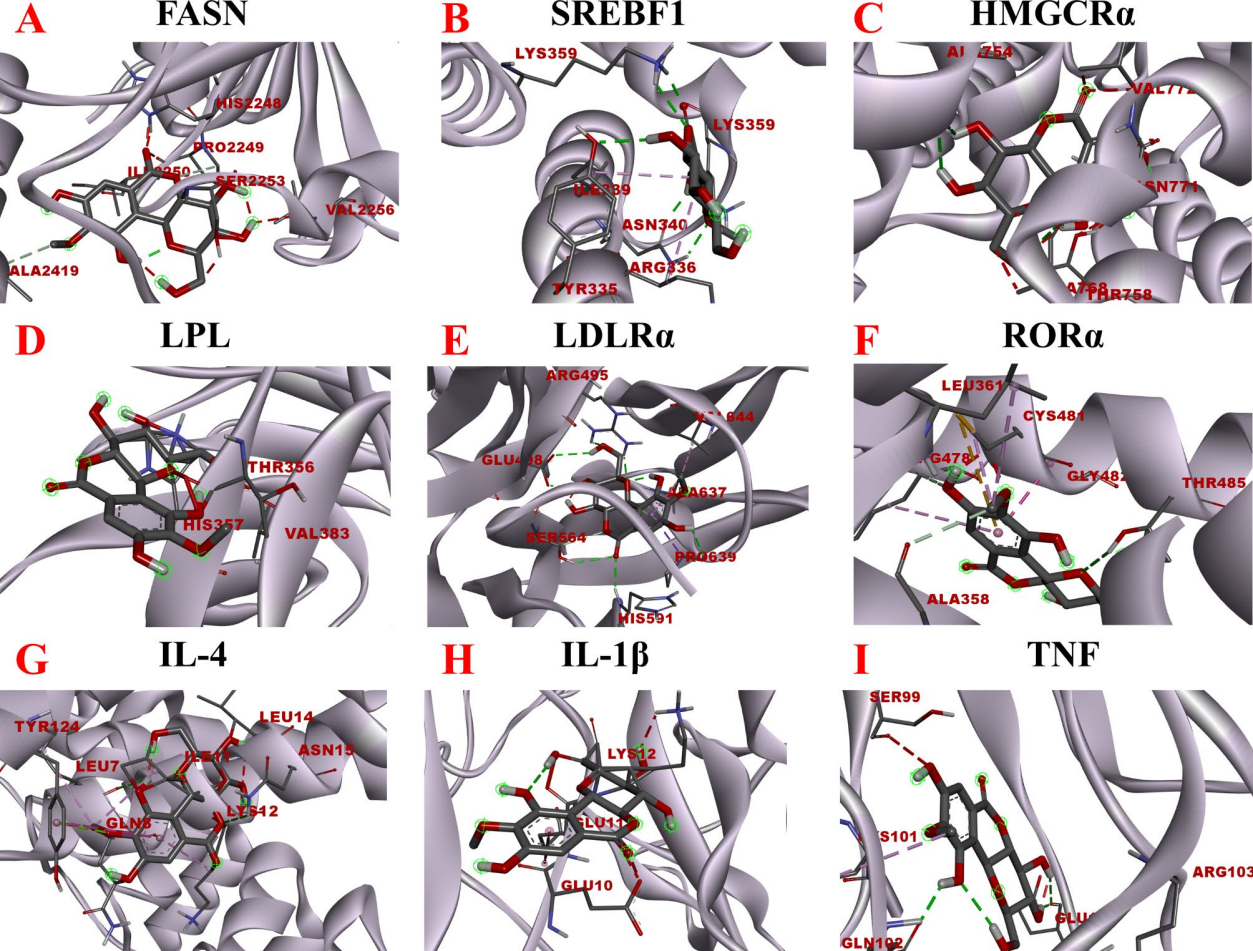
Molecular docking visual analysis of bergenin binding to FASN, SREBF1, HMGCRα, LPL, LDLRα, RORα, IL-4, IL-1β, and TNF. (**A**), (**B**), (**C**), (**D**), (**E**), (**F**), (**G**), (**H**), and (**I**) correspond to the binding models of bergenin with FASN, SREBF1, HMGCRα, LPL, LDLRα, RORα, IL-4, IL-1β, and TNF, respectively

Molecular docking is a technique used to identify the active site where a small molecule ligand interacts with a target protein molecule, allowing them to form a stable and energetically favorable binding conformation. The binding energy value reflects the strength of the interaction between the compound and the protein receptor, with lower binding energy values indicating higher binding activity [54]. To corroborate the outcomes of the RT‒PCR experiments, molecular docking was employed to visualize the binding interactions of bergenin with its key protein targets, as illustrated in Fig. 9A-I. Bergenin docks into a position analogous to the original ligand, occupying the active pocket formed by the amino acid residues of the protein. The binding energies of bergenin to the eight target receptors (FASN, SREBF1, HMGCRα, RORα, LDLRα, IL-4, IL-1β, and TNF) were all ≤ -6.0 kcal/mol, as indicated in Table 2. Specifically, the binding energies of bergenin (Fr2) to IL-4, LDLRα, and RORα were − 7.53, -7.48, and − 7.10 kcal/mol, respectively, indicating their strong binding activity, as the values were lower than − 7.0 kcal/mol. Expanding on this, the potent binding interactions between bergenin and IL-4 are primarily facilitated through robust hydrogen bonding interactions with LEU-7 and ASN-15 amino acid residues, along with the formation of Pi-alkyl, electrostatic, and π-π interactions with LEU-7, LEU-14, ILE-11, TYR-124, and LYS-12, residues. The interactions between bergenin and LDLRα exhibit Pi-Sigma interactions with PRO-639 residues, in addition to hydrogen bonding (PRO-639, HIS-591, SER-564, ARG-495, GLU-498, ALA-637) and Pi-alkyl (VAL-644, ALA-637) forces with the residues. Similarly, hydrogen bonding (THR-485, GLY-482, ARG-478, ALA-358) and Pi-alkyl (ARG-478, CYS-481, LEU-361) forces are formed between bergenin and RORα with the residues. Conversely, bergenin demonstrated a binding energy of only − 4.06 kcal/mol with the LPL protein. A docking binding energy below − 5.0 kcal/mol suggests that the compound exhibits some binding activity with the target protein, while a docking binding energy below − 7 kcal/mol indicates robust binding activity between the compound and the target protein.

**Table 2:** Intermolecular interactions between bergenin and FASN, SREBF1, HMGCRα, LPL, LDLRα, RORα, IL-4, IL-1β, and TNF

Proteins	Binding Energy (kcal/mol)	Binding Residues	Type
FASN	-6.56	ALA-2419 SER-2253 VAL-2256 PRO-2249 HIS-2248 ILE-2250	Conventional Hydrogen Bond Pi-Sigma Carbon Hydrogen Bond Conventional Hydrogen Bond Conventional Hydrogen Bond Conventional Hydrogen Bond
SREBF1	-6.15	LYS-359 ASN-340 ARG-336 ILE-339 LYS-359 TYR-335	Conventional Hydrogen Bond Conventional Hydrogen Bond Pi-Alkyl Pi-Alkyl Conventional Hydrogen Bond Conventional Hydrogen Bond
HMGCRα	-6.09	ALA-754 VAL-772 ALA-768 ALA-768 ASN-771 THR-758	Conventional Hydrogen Bond Carbon Hydrogen Bond Carbon Hydrogen Bond Pi-Alkyl Conventional Hydrogen Bond Pi-Anion
LPL	-4.06	THR-356 VAL-383 HIS-357 HIS-357	Conventional Hydrogen Bond Conventional Hydrogen Bond Carbon Hydrogen Bond Pi-Anion
LDLRα	-7.48	PRO-639 PRO-639 VAL-644 HIS-591 SER-564 SER-564 ALA-637 ALA-637 ARG-495 GLU-498	Conventional Hydrogen Bond Pi-Sigma Pi-Alkyl Conventional Hydrogen Bond Conventional Hydrogen Bond Carbon Hydrogen Bond Carbon Hydrogen Bond Pi-Alkyl Conventional Hydrogen Bond Conventional Hydrogen Bond
RORα	-7.10	ARG-478 ARG-478 CYS-481 LEU-361 ALA-358 GLY-482 THR-485	Carbon Hydrogen Bond Pi-Alkyl Pi-Alkyl Pi-Alkyl Carbon Hydrogen Bond Carbon Hydrogen Bond Conventional Hydrogen Bond
IL-4	-7.53	LEU-7 LEU-7 LEU-14 ILE-11 TYR-124 TYR-124 TYR-124 LYS-12 GLN-8 ASN-15	Conventional Hydrogen Bond Pi-Alkyl Pi-Alkyl Pi-Alkyl Pi-Alkyl Pi-Pi T-shaped Pi-Lone Pair Pi-Alkyl Pi-Lone Pair Conventional Hydrogen Bond
IL-1β	6.18	LYS-12 LYS-12 GLU-11 GLU-10 GLU-10	Conventional Hydrogen Bond Carbon Hydrogen Bond Carbon Hydrogen Bond Pi-Alkyl Amide-Pi Stacked
TNF	-6.87	SER-99 CYS-101 GLN-102 GLN-102 ARG-103 GLU-110	Conventional Hydrogen Bond Pi-Alkyl Conventional Hydrogen Bond Carbon Hydrogen Bond Conventional Hydrogen Bond Conventional Hydrogen Bond

Therefore, the molecular docking results of bergenin with these target proteins are consistent with the findings from the qRT‒PCR experiments conducted in the zebrafish model. In the in vivo experiments investigating the effects of bergenin on high-fat zebrafish, the qRT‒PCR results revealed that a low concentration (5 μM) of bergenin effectively regulated the mRNA expression of IL-4, LDLRα, and RORα. Furthermore, a high concentration (15 μM) of bergenin effectively regulated the expression of FASN, SREBF1, HMGCRα, IL-1β, and TNF mRNA. However, neither low nor high concentrations of bergenin showed a significant effect on the expression of LPL mRNA. Molecular docking serves to explore the theoretical level of specific action modes and binding conformations between small drug molecules and macromolecular targets, while qRT‒PCR experiments in the zebrafish model investigate the actual gene level. The mutual validation of these two approaches provides a scientific basis for understanding the mechanism of action of bergenin against hyperlipidemia. In conclusion, this study showed that bergenin exerts an effect on lipid metabolism in zebrafish fed a high-fat diet, and its mechanism of action may involve SREBF1-, FASN-, and RORα-related signaling pathways. However, further investigation is needed to elucidate the specific molecular mechanisms underlying the action of bergenin.

## Conclusion

In this study, the dried entire herb of *S. melanocentra* was extracted using methanol, and the resulting crude extract was combined with polyamide. Through a two-repetition separation process, the major component bergenin was successfully isolated from the dried mixture using a one-step polyamide MPLC contemporary chromatographic procedure. The purity of bergenin (Fr2) was confirmed to be over 98% through verification with RP columns and HILIC columns. To explore its medicinal potential further, bergenin, the primary component, was investigated for its pharmacological effects against atherosclerosis in this study. Bergenin treatment at varying doses significantly reduced TC, TG, and LDL-c activities while increasing HDL-c in hyperlipidemic zebrafish. Oil Red O staining results further revealed a substantial improvement in liver damage in hyperlipidemic zebrafish, with the degree of improvement showing a dose-dependent pattern. This finding implies that bergenin preserves the structural integrity of hepatocytes and facilitates the repair of liver injuries induced by a high-fat diet. Moreover, the assessment of mRNA expression levels of relevant proteins demonstrated that bergenin ameliorates lipid metabolism abnormalities by inhibiting the expression of RORα, modulating the expression of HMGCRα, LDLRα, IL-4, IL-1β, and TNF, and subsequently restraining cholesterol synthesis. This multifaceted approach reduces the inflammatory response within the body, contributing to the hypolipidemic effects observed. Molecular docking techniques were employed to gain insights into its potential hypolipidemic mechanism, which appears to involve SREBF1, FASN, and RORα-related signaling pathways, leading to the inhibition of lipid and cholesterol synthesis and a reduction in the inflammatory response. The findings of our study provide a robust foundation for understanding the hypolipidemic action of bergenin, suggesting its potential development into a mild, efficient, and safe hypolipidemic product. It is important to note that, to date, only the in vivo hypolipidemic activity and the associated gene-level performance of bergenin have been confirmed. If this compound is to be advanced into drug products, further comprehensive investigations are warranted to explore aspects such as targeting mechanisms, stability, bioavailability, and other parameters influencing its hypolipidemic effects. Increased experimentation in the preclinical research process will be essential for a more thorough understanding of its potential applications.

## Data Availability

All data generated or analyzed during this study are included in this published article.

## Abbreviations

Ber: Bergenin
Ffb: Fenofibrate
HDL-c: High-density lipoprotein cholesterol
HILIC: Hydrophilic interaction chromatography
LDL-c: Low-density lipoprotein cholesterol
MPLC: Medium-pressure liquid chromatography
NPs: Natural products
RPLC: Reversed-phase liquid chromatograph
*S. melanocentra*: *Saxifraga melanocentra* Franch
TG: Triglyceride
TC: Total cholesterol

## References

[1] Bozkurt B, Aguilar D, Deswal A, Dunbar SB, Francis GS, Horwich T, Jessup M, Kosiborod M, Pritchett AM, Ramasubbu K, Rosendorff C, Yancy C (2016). American heart association Heart Failure and transplantation committee of the council on clinical cardiology; council on cardiovascular Surgery and anesthesia; council on cardiovascular and Stroke nursing; council on Hypertension; and council on quality and outcomes research. Contributory risk and management of comorbidities of Hypertension, obesity, Diabetes Mellitus, hyperlipidemia, and metabolic syndrome in chronic Heart Failure: a scientific statement from the American heart association. Circulation.

[2] Parhofer KG, Laufs U (2019). The diagnosis and treatment of Hypertriglyceridemia. Deutsches Arzteblatt International.

[3] Benchoula K, Khatib A, Jaffar A, Ahmed QU, Sulaiman WMAW, Abd Wahab R, El-Seedi HR (2019). The promise of zebrafish as a model of metabolic syndrome. Exp Anim.

[4] Fang LH, Miller YI (2012). Emerging applications for zebrafish as a model organism to study oxidative mechanisms and their roles in inflammation and vascular accumulation of oxidized lipids. Free Radic Biol Med.

[5] Wang WY, Yan ZH, Yao HL, Li PB, Peng W, Su WW, Wang YG (2021). Extraction and purification of pedunculoside from the dried barks of *Ilex rotunda* using crystallization combined with polyamide column chromatography. Sep Sci Technol.

[6] Háková M, Havlíková LC, Chvojka J, Erben J, Solich P, Švec F, Šatínský D (2018). A comparison study of nanofiber, microfiber, and new composite nano/microfiber polymers used as sorbents for on-line solid phase extraction in chromatography system. Anal Chim Acta.

[7] Radlmaier V, Heckel C, Winnacker M, Erber A, Koerber H (2017). Effects of thermal cycling on polyamides during processing. Thermochimica Acta.

[8] Kim NH, Kim SG (2020). Fibrates revisited: potential role in Cardiovascular Risk reduction. Diabetes & Metabolism Journal.

[9] Mitchell JP, Carmody RJ (2018). NF-κB and the Transcriptional Control of Inflammation. Int Rev Cell Mol Biology.

[10] Thompson PD, Panza G, Zaleski A, Taylor B (2016). Statin-Associated Side effects. J Am Coll Cardiol.

[11] Li JF, Liu ZL (2022). Complete chloroplast genome sequence of *Micranthes melanocentra* (Saxifragaceae). Mitochondrial DNA Part B.

[12] Li Y, Li F, Zheng TT, Shi L, Zhang ZG, Niu TM, Wang QY, Zhao DS, Li W, Zhao P (2021). Lamiophlomis herba: a comprehensive overview of its chemical constituents, pharmacology, clinical applications, and quality control. Biomed Pharmacother.

[13] Zhang XX, Zhan GQ, Jin M, Zhang H, Dang J, Zhang Y, Guo ZJ, Ito Y (2018). Botany, traditional use, phytochemistry, pharmacology, quality control, and authentication of Radix Gentianae Macrophyllae-A traditional medicine: a review. Phytomedicine.

[14] Li XH, Wang YH, Liang JJ, Bi Z, Ruan H, Cui YY, Ma L, Wei YL, Zhou BC (2021). Bergenin attenuates bleomycin-induced pulmonary fibrosis in mice via inhibiting TGF‐β1 signaling pathway. Phytother Res.

[15] Xiang SH, Chen K, Xu L, Wang T, Guo CY (2020). Bergenin exerts Hepatoprotective effects by inhibiting the release of inflammatory factors, apoptosis and autophagy via the PPAR-γ pathway. Drug Des Devel Ther.

[16] Bajracharya GB (2015). Diversity, pharmacology and synthesis of bergenin and its derivatives: potential materials for therapeutic usages. Fitoterapia.

[17] El-Hawary SS, Mohammed R, Abouzid S, Ali ZY, Elwekeel A (2016). Anti-arthritic activity of 11-O-(4’-O-methyl galloyl)-bergenin and Crassula capitella extract in rats. J Pharm Pharmacol.

[18] Torres PHM, Sodero ACR, Jofily P, Silva-Jr FP (2019). Key topics in Molecular Docking for Drug Design. Int J Mol Sci.

[19] Śledź P, Caflisch A (2018). Protein structure-based drug design: from docking to molecular dynamics. Curr Opin Struct Biol.

[20] Afroz M, Zihad SMNK, Uddin SJ, Rouf R, Rahman MS, Islam MT, Khan IN, Ali ES, Aziz S, Shilpi JA, Nahar L, Sarker SD (2019). A systematic review on antioxidant and antiinflammatory activity of Sesame (*Sesamum indicum* L.) oil and further confirmation of antiinflammatory activity by chemical profiling and molecular docking. Phytother Res.

[21] Rosell M, Fernández-Recio J (2020). Docking approaches for modeling multi-molecular assemblies. Curr Opin Struct Biol.

[22] Dang J, Ma JB, Du YR, Dawa YZ, Wang Q, Chen CB, Wang QL, Tao YD, Ji TF (2021). Large-scale preparative isolation of bergenin standard substance from *Saxifraga atrata* using polyamide coupled with MCI GEL® CHP20P as stationary phases in medium pressure chromatography. J Chromatogr B.

[23] Kumar R, Patel DK, Prasad SK, Laloo D, Krishnamuthy S, hemalatha S (2012). Type 2 antidiabetic activity of bergenin from the roots of Caesalpinia Digyna Rottler. Fitoterpia.

[24] Bleau H, Daniel C, Chevalier G, vanTra H, Hontela A (1996). Effects of acute exposure to mercury chloride and methylmercury on plasma cortisol, T3, T4, glucose and liver glycogen in rainbow trout (*Oncorhynchus mykiss*). Aquat Toxicol.

[25] Lee YJ, Choi HS, Seo MJ, Jeon HJ, Kim KJ, Lee BY (2015). Kaempferol suppresses lipid accumulation by inhibiting early adipogenesis in 3T3-L1 cells and zebrafish. Food Funct.

[26] Caro M, Sansone A, Amezaga J, Navarro V, Ferreri C, Tueros I (2017). Wine lees modulate lipid metabolism and induce fatty acid remodelling in zebrafish. Food Funct.

[27] Suh HJ, Cho SY, Kim EY, Choi HS (2015). Blockade of lipid accumulation by silibinin in adipocytes and zebrafish. Chemico-Biol Interact.

[28] Zhang YI, Li X, Liu ZT, Zhao XY, Chen L, Hao GJ, Ye XP, Meng SL, Xiao GH, Mu JD, Mu XY (2023). The neurobehavioral impacts of typical antibiotics toward zebrafish larvae. Chemosphere.

[29] Ding YR, Xiao C, Wu QP, Xie YZ, Li XM, Hu HP, Li LQ. The mechanisms underlying the Hypolipidaemic effects of Grifola frondosa in the liver of rats. Front Microbiol. 2016;7. 10.3389/fmicb.2016.01186 10.3389/fmicb.2016.01186 PMC4971090 27536279

[30] Hooft F, Ortiz APD, Ensing B (2021). Discovering Collective Variables of Molecular Transitions via Genetic Algorithms and neural networks. J Chem Theory Comput.

[31] Piri-Moghadam H, Alam MN, Pawliszyn J (2017). Review of geometries and coating materials in solid phase microextraction: opportunities, limitations, and future perspectives. Anal Chim Acta.

[32] Wang Q, Chen WJ, Wang QL, Tao YD, Yu RT, Pan GQ, Dang J (2020). Preparative separation of isoquinoline alkaloids from *Corydalis impatiens* using middle chromatogram isolated gel column coupled with positively charged reversed-phase liquid chromatography. J Sep Sci.

[33] Zhao YN, Li HZ, Zhang ZJ, Ren ZQ, Yang FH (2022). Extraction, preparative monomer separation and antibacterial activity of total polyphenols from Perilla frutescens. Food Funct.

[34] Erben J, Klicova M, Klapstova A, Háková M, Lhotská I, Zatrochová S, Šatínský D, Chvojka J (2022). New polyamide 6 nanofibrous sorbents produced via alternating current electrospinning for the on-line solid phase extraction of small molecules in chromatography systems. Microchem J.

[35] Liu RJ, Kool J, Jian JY, Wang JC, Zhao XL, Jiang ZJ, Zhang TT (2021). Rapid Screening α-Glucosidase inhibitors from Natural products by At-Line nanofractionation with parallel Mass Spectrometry and Bioactivity Assessment. J Chromatogr A.

[36] Libby P, Buring JE, Badimon L, Hansson GK, Deanfield J, Bittencourt MS, Tokgözoğlu L, Lewis EF, Atherosclerosis (2019). Nat Reviews Disease Primers.

[37] Oikonomou EK, Antoniades C (2019). The role of adipose tissue in cardiovascular health and Disease. Nat Reviews Cardiol.

[38] McCalley DV (2017). Understanding and manipulating the separation in hydrophilic interaction liquid chromatography. J Chromatogr A.

[39] Wang D, Zhu HT, Zhang YJ, Yang CR, Bioorganic (2005). Med Chem Lett.

[40] Song ZY, Xiaoli AM, Yang FJ (2018). Regulation and metabolic significance of *De Novo* Lipogenesis in adipose tissues. Nutrients.

[41] Shahinfar H, Bazshahi E, Amini MR, Payandeh N, Pourreza S, Noruzi Z, Shab-Bidar S (2021). Effects of artichoke leaf extract supplementation or artichoke juice consumption on lipid profile: a systematic review and dose-response meta-analysis of randomized controlled trials. Phytother Res.

[42] Nakamura MT, Yudell BE, Loor JJ (2014). Regulation of energy metabolism by long-chain fatty acids. Prog Lipid Res.

[43] Wu Q, Yan W, Liu CS, Li L, Yu LQ, Zhao SJ, Li GY (2016). Microcystin-LR exposure induces developmental neurotoxicity in zebrafish embryo. Environ Pollut.

[44] Goundadkar BB, Katti P (2017). Environmental estrogen(s) induced swimming behavioural alterations in adult zebrafish (*Danio rerio*). Environ Toxicol Pharmacol.

[45] dos Santos KPE, Silva IF, Mano-Sousa BJ, Duarte-Almeida JM, de Castro WV, Ribeiro RIMD, Santos HB, Thom RG. Abamectin promotes behavior changes and liver injury in zebrafish. Chemosphere. 2023;311(1). 10.1016/j.chemosphere.2022.136941 10.1016/j.chemosphere.2022.136941 36272627

[46] Yan J, Horng T (2020). Lipid metabolism in regulation of macrophage functions. Trends Cell Biol.

[47] Shapouri-Moghaddam A, Mohammadian S, Vazini H, Taghadosi M, Esmaeili SA, Mardani F, Seifi B, Mohammadi A, Afshari JT, Sahebkar A (2018). Macrophage plasticity, polarization, and function in health and Disease. J Cell Physiol.

[48] Lan JR, Zhao YY, Dong FX, Yan ZY, Zheng WJ, Fan JP, Sun GL (2015). Meta-analysis of the effect and safety of berberine in the treatment of type 2 Diabetes Mellitus, hyperlipemia and Hypertension. J Ethnopharmacol.

[49] Luo J, Yang HY, Song BL (2020). Mechanisms and regulation of cholesterol homeostasis. Nat Rev Mol Cell Biol.

[50] Ahmadi Y, Ghorbanihaghjo A, Argani H (2017). The balance between induction and inhibition of mevalonate pathway regulates cancer suppression by statins: a review of molecular mechanisms. Chemico-Biol Interact.

[51] Bridgewood C, Newton D, Bragazzi N, Wittmann M, McGonagle D (2021). Unexpected connections of the IL-23/IL-17 and IL-4/IL-13 cytokine axes in inflammatory arthritis and enthesitis. Semin Immunol.

[52] Feltrin S, Ravera F, Traversone N, Ferrando L, Bedognetti D, Ballestrero A, Zoppoli G (2020). Sterol synthesis pathway inhibition as a target for cancer treatment. Cancer Lett.

[53] Kalliolias GD, Ivashkiv LB (2016). TNF biology, pathogenic mechanisms and emerging therapeutic strategies. Nat Rev Rheumatol.

[54] Genheden S, Ryde U (2015). The MM/PBSA and MM/GBSA methods to estimate ligand-binding affinities. Expert Opin Drug Discov.

